# Is There a Typho-Malarial Fever?

**Published:** 1896-11

**Authors:** 


### Abstracts and Selections.

### Is There a Typho-Malarial Fever?

The Secretary reported a case not because of uniqueness, but as contributing to the settlement of much mooted existence of typho-malarial fever.

T. H. P., male, age 36, more than three weeks ago was taken sick with what looked like cholera morbus. In four days a chill came on followed by sweating and fever; thereafter, for four or five days, there were two and three chills at irregular intervals daily; the temperature one morning reaching 106°, falling to 102° upon the application of a cold bath. Thirty grains of hydrochlorate of quinine were administered in twelve hours, and as a result chills ceased, but there remained a persistent fever, which was stationary in the morning, rising in the afternoon, and accompanied by nearly all the symptoms which justified a diagnosis of typhoid fever. He entered his fourth week of sickness to-day, but his temperature has been normal for four or five days in the morning, with exacerbation in the afternoon. Chills came on again last Friday, and with the exception of Sunday, has had them daily at irregular intervals, and sometimes two in twenty-four hours.

Dr. R. F. Williams made a blood examination and found absolutely no plasmodium, but a marked leucocytosis which argues a favorable result.

Drs. Gordon and Deaton saw the case in consultation, and agreed that the trouble is pure typhoid.

Dr. Wm. S. Gordon said, regarding this case, that it was undoubtedly typhoid. If it had been typho-malaria, when the chills were stopped, the fever should have ceased, and the blood examination further verified the diagnosis. He reported the case of a lady confined two weeks ago. He had ceased his attention, when on the twelfth day, she was seized by a violent chill at 4:30 p. m., and again at 11 p. m. Friday last there was another at 11 a. m. Quartan fever being suspected, he gave quinine, but there ensued a chill at 11 p. m., followed by high fever and sweating. There was no trouble with the breast that morning; the lochia were free from odor, and everything relating to the uterus was normal. At night, the breast was affected and lead-water was advised. The next morning mammitis had come on--no suppuration. The breast trouble did not ensue until the fourth day. It was relieved. Yesterday, the patient was entirely free from fever. She was thoroughly cinchonized. Remembering the influence on the mind of the time of attacks, he endeavored to take her's from under its influence by mental diversion, anecdotes, etc.; but at 4:30 p. m. the chill came. If she had not been under the influence of quinine, she would in all probability have had another last night. The case, a double quotidian of the quartan type, is one the doctor has never seen before.

As to the cause: The house is surrounded by flowers, grown over with vines; has, necessarily, decaying vegetation in the garden; flowers in the rooms. These, with moisture and heat, furnish all the etiology desired.—*Trans: Richmond Academy of Medicine.*

